# Abundance and Seasonality of *Aedes aegypti* (Diptera: Culicidae) in Two Suburban Localities of South Mexico, With Implications for *Wolbachia* (Rickettsiales: Rickettsiaceae)-Carrying Male Releases for Population Suppression

**DOI:** 10.1093/jme/tjab052

**Published:** 2021-04-02

**Authors:** Azael Che-Mendoza, Abdiel Martin-Park, Juan Manuel Chávez-Trava, Yamili Contreras-Perera, Hugo Delfín-González, Gabriela González-Olvera, Jorge Leirana-Alcocer, Guillermo Guillermo-May, Daniel Chan-Espinoza, Norma Pavia-Ruz, Rosa Eugenia Méndez-Vales, Alberto Alcocer-Gamboa, Fabian Correa-Morales, Jorge Palacio-Vargas, Dongjing Zhang, Gonzalo Vazquez-Prokopec, Zhiyong Xi, Pablo Manrique-Saide

**Affiliations:** 1 Laboratorio para el Control Biológico de Aedes aegypti (LCB-UADY), Unidad Colaborativa para Bioensayos Entomológicos (UCBE), Campus de Ciencias Biológicas y Agropecuarias, Universidad Autónoma de Yucatán, Km. 15.5 Carr. Mérida-Xmatkuil s.n., Mérida, Yucatán C.P. 97315, México; 2 Departamento de Ecología, Campus de Ciencias Biológicas y Agropecuarias, Universidad Autónoma de Yucatán, Km. 15.5 Carr. Mérida-Xmatkuil s.n., Mérida, Yucatán C.P. 97315, México; 3 Centro de Investigaciones Regionales, Dr. Hideyo Noguchi, Universidad Autónoma de Yucatán, Calle 59 x Itzáes Avenue, Centro, C.P. 97000, Mérida, Yucatán, México; 4 Servicios de Salud de Yucatán, Calle 72 #463 por 53 y 55 C.P. 97000, Mérida, Yucatán, México; 5 Secretaría de Investigación, Innovación y Educación Superior, Calle 8 347, San Esteban, C.P. 97149 Mérida, Yucatán, México; 6 Subdirección del Programa de Enfermedades Transmitidas por Vectores, Centro Nacional de Programas Preventivos y Control de Enfermedades, Benjamín Franklin No. 132, Col. Escandón Del. Miguel Hidalgo, C.P. 11800, México,México; 7 Sun Yat-sen University–Michigan State University Joint Center of Vector Control for Tropical Diseases, Guangzhou 510080, China; 8 Department of Environmental Sciences, Emory University, 400 Dowman Dr, 5th Fl, Ste E523, Atlanta, GA, 30322, USA; 9 Department of Microbiology and Molecular Genetics, Michigan State University, East Lansing, MI 48824, USA

**Keywords:** *Aedes aegypti*, incompatible insect technique, sterile insect technique, *Wolbachia*, population suppression

## Abstract

We conducted a baseline characterization of the abundance and seasonality of *Aedes aegypti* (Linnaeus, 1762)—a vector of dengue, chikungunya, and Zika—in two suburban localities of Yucatan, Mexico, as the first step in the implementation of an integrated vector management (IVM) plan combining ‘traditional *Aedes* control’ (source reduction/truck-mounted ultra-low volume [ULV] spraying) and incompatible insect technique/sterile insect technique for population suppression in Yucatan, Mexico. Weekly entomological collections with ovitraps and BG-sentinel traps were performed in 1-ha quadrants of both localities for 1 yr. Three distinct periods/phases were identified, closely associated with precipitation: 1) a phase of low population abundance during the dry season (weekly average of *Aedes* eggs per ovitrap and adults per BG trap = 15.51 ± 0.71 and 10.07 ± 0.88, respectively); 2) a phase of population growth and greatest abundance of *Aedes* (49.03 ± 1.48 eggs and 25.69 ± 1.31 adults) during the rainy season; and finally 3) a phase of decline among populations (20.91 ± 0.97 eggs and 3.24 ± 0.21 adults) after the peak of the rainy season. Seasonal abundance and dynamics of *Ae. aegypti* populations suggest that it is feasible to develop and implement time-specific actions as part of an IVM approach incorporating integrating novel technologies (such as rear-and-release of *Wolbachia*-infected males) with classic (insecticide-based) approaches implemented routinely for vector control. In agreement with the local vector control program, we propose a pilot IVM strategy structured in a preparation phase, an attack phase with traditional vector control, and a suppression phase with inundative releases, which are described in this paper.

‘Rear-and-release’ of mosquitoes for population suppression is gaining interest and recognition as an innovative approach with potential for successful control of *Aedes aegypti*, the main vector of dengue, chikungunya, and Zika (Pan American Health Organization 2019, World Health Organization and International Atomic Energy Agency 2020). Initiatives using the sterile insect technique (SIT) by irradiation and/or the incompatible insect technique (IIT), involving sequential inundative releases of mass-produced male mosquitoes, are currently under preparation and/or implementation in multiple countries (Kittayapong et al. 2019, Mains et al. 2019, Bouyer et al. 2020, Crawford et al. 2020).

In 2016, the government of the Mexican state of Yucatan signed an international collaboration agreement with Michigan State University (MSU) and the Autonomous University of Yucatan (UADY) for the development and application of strategies based on a combination of IIT and SIT (Zheng et al. 2019) to suppress *Ae. aegypti* populations. This approach involves the production and release of mass-reared *Aedes* males from a mosquito line infected with *Wolbachia w*AlbB (Xi et al. 2005) that are irradiated with X-rays in the pupal stage. Due to cytoplasmic incompatibility (CI), wild-type female *Ae. aegypti* mating with released males will produce infertile eggs. Furthermore, any *Wolbachia*-carrying female accidentally released will not be able to reproduce, minimizing any undesirable establishment of *Wolbachia* in the wild. The *w*AlbB strain of *Wolbachia* is of relevance for IIT, as it has shown its stable and strong CI in *Ae. aegypti*, with minimal effects on mosquito fitness (Xi et al. 2005, Axford et al. 2016). Given that *w*AlbB in *Ae. aegypti* does not affect male mating success (Axford et al. 2016) and shows great stability at high temperatures (Ross et al. 2017), it has been suggested to be a suitable option for population replacement/suppression in warm climates (Nazni et al. 2019).

Baseline studies to describe the abundance and phenology of *Ae. aegypti* populations are an initial requirement prior to develop field pilot tests at any potential study site (Pan American Health Organization 2019, Bouyer et al. 2020, World Health Organization and International Atomic Energy Agency 2020). In this context, mosquito surveillance is an essential requisite for monitoring and evaluation of future mosquito releases (World Health Organization and International Atomic Energy Agency 2020). Two entomological surveillance methods have been commonly performed for assessment of population suppression: the deployment of oviposition traps to monitor the abundance of populations and effective expression of *Wolbachia* induced-CI retrieved eggs, and the use of BG-sentinel traps to estimate the infestation levels of adult *Ae. aegypti* (Zheng et al. 2019, World Health Organization and International Atomic Energy Agency 2020).

Here, we report results of a study that collected baseline entomological data on the abundance and seasonal variation of *Ae. aegypti* in two suburban localities of south Mexico and discuss our results in the context of the future implementation, in coordination with the Vector Control Program of the Ministry of Health of Yucatan, Mexico, of an integrated vector management (IVM) plan combining ‘traditional *Aedes* control’ (source reduction/truck-mounted ultra-low volume [ULV] spraying) and IIT/SIT for population suppression (project ‘Integrated vector management plan based on use of *Wolbachia*-carrying male mosquitoes for population suppression of *Aedes aegypti*’).

## Materials and Methods

### Study Sites

**Fig. 1.**
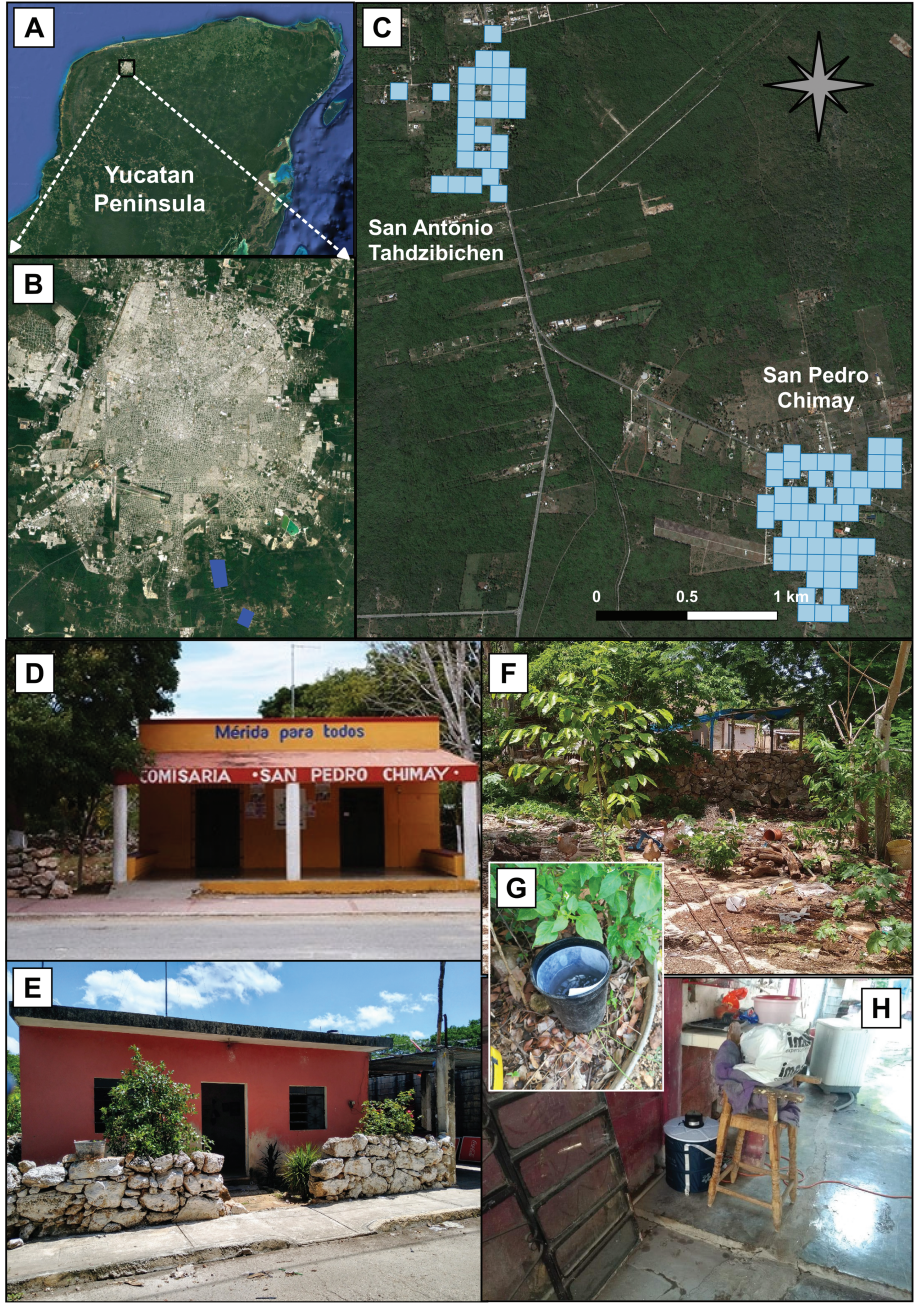
(A–C) Location of the study sites and sampled areas (in blue); (D–F) example of housing and backyards, (G) ovitrap, and (H) BG-sentinel trap employed for collections.

San Pedro Chimay (SPC) and San Antonio Tahdzibichen (TAH), two suburban localities in the periphery of the city of Merida, Mexico (Fig. 1A and B), were selected for a pilot or initial implementation study of the combined traditional *Wolbachia* IIT/SIT control strategy. The selection of both towns was done in consensus with the Ministry of Health (MoH) of Yucatan, that considered them suitable because they are 1) geographically isolated, 2) small (30–50 Ha), 3) suburban towns with similar sociodemographic and ecological environments for *Ae. aegypti*, and (4) with no active arbovirus transmission.

SPC has a human population of 1,241 inhabitants distributed across 300 houses (46.2 ha). TAH has a population of 724 individuals across 174 houses (30 ha) (Instituto Nacional de Estadística y Geografía 2019). Localities are isolated by local vegetation and separated 3.3 km from each other. The average altitude of the localities is 9 m above sea level. The climate is mainly warm with an annual average temperature of 26.3°C (34.2°C max and 18.4°C min), with two distinct annual phases: a rainy season, from May/June to October with a rainfall of 990.6 mm, and a dry season from November to April with rainfall of 291.2 mm (Instituto Nacional de Estadística y Geografía 2019).

### Entomological Collections

We focused our monitoring plan on detecting *Ae. aegypti* eggs and adults, which are relevant life stages for entomological surveillance of the IIT/SIT method, as described by previous pilot studies using the population suppression approach (Kittayapong et al. 2019, Mains et al. 2019, Zheng et al. 2019, Crawford et al. 2020). Monitoring of *Ae. aegypti* populations at both study sites was conducted by well-trained and experienced field staff from the MoH and the Collaborative Unit for Entomological Bioassays (UCBE-UADY) during 2017. The study sites were divided into 1-ha areas (Fig. 1B), each with four to eight houses, to establish sentinel sampling stations for entomological surveillance.

### Oviposition Traps

Oviposition was monitored weekly with 100 ovitraps per locality (≈2 per ha) from January to December 2017. Standard ovitraps employed by the Mexican MoH (Centro Nacional de Programas Preventivos y Control de Enfermedades 2015), which consist of a 1-liter black plastic container covered in its upper third with a strip of fabric cloth (F-1600) as substrate for oviposition (ovistrip), were placed with water (three fourth of its capacity) in the peridomicile (exterior) of the houses (Fig. 1D). Ovistrips were collected weekly and transported to UCBE-UADY, according to the Mexican guidelines for embryogenesis, storage, and shipment (Centro Nacional de Programas Preventivos y Control de Enfermedades 2015). The number of eggs was determined by visual examination using a stereomicroscope (Olympus).

### Adult Collections

To monitor the adult mosquito population (outdoors), we set one BG trap (Biogents) with an octenol-based attractant (Octenol Mosquito Magnet) (Fig. 1E) in the near-outer surrounding of randomly selected houses with ovitraps (SPC *n* = 25 and TAH *n* = 25) from May to December. The collections included one 24-h cycle every week. All mosquitoes collected were identified for species and sex. The number of males and females in each trap was counted and recorded.

### Databases and Analysis

From ovitraps, we calculated the egg density index (EDI) = total number of eggs/numbers of ovitraps. From outdoor adult collections with BG traps, we calculated: 1) adult density index (ADI) = total number of adult *Aedes*/traps (in a premise), and derived calculations of 2) ADI for male *Aedes*, 3) ADI for female *Aedes*, and 4) ADI for blood-fed females collected in 24-h cycles.

We present a descriptive analysis of the temporal variation in the different entomological indicators per week/month and temperature and rainfall (Comisión Nacional del Agua 2020). Cross-correlation function (CCF) and Wilcoxon signed-rank tests were carried out to compare 1) seasonality (i.e., cross-correlation of mean weekly eggs collected between localities and between egg collections and mean daily rainfall for each week, considering different lag of weeks for rain) and 2) differences between medians of mosquito abundance by season between SPC versus TAH, respectively. All the analyses were run with the software platform R (https://www.r-project.org/).

## Results

### Abundance and Seasonality of Populations

No significant differences in the median *Aedes* eggs were found between SPC and TAH before, during, or after the rainy season (Wilcoxon signed-rank test: *W* = 72, *P* = 0.32; *W* = 1,029, *P* = 0.32; and *W* = 48, *P* = 0.47, respectively). The abundance of *Ae. aegypti* adult females (*W* = 3, *P* = 0.16; *W* = 1,078, *P* = 0.88; and *W* = 48, *P* = 0.52) and males (*W* = 3, *P* = 0.16; *W* = 1,077, *P* = 0.34; and *W* = 48, *P* = 0.48) was comparable between both localities in each rainy period (before, during, and after the rainy season, respectively).

**Fig. 2.**
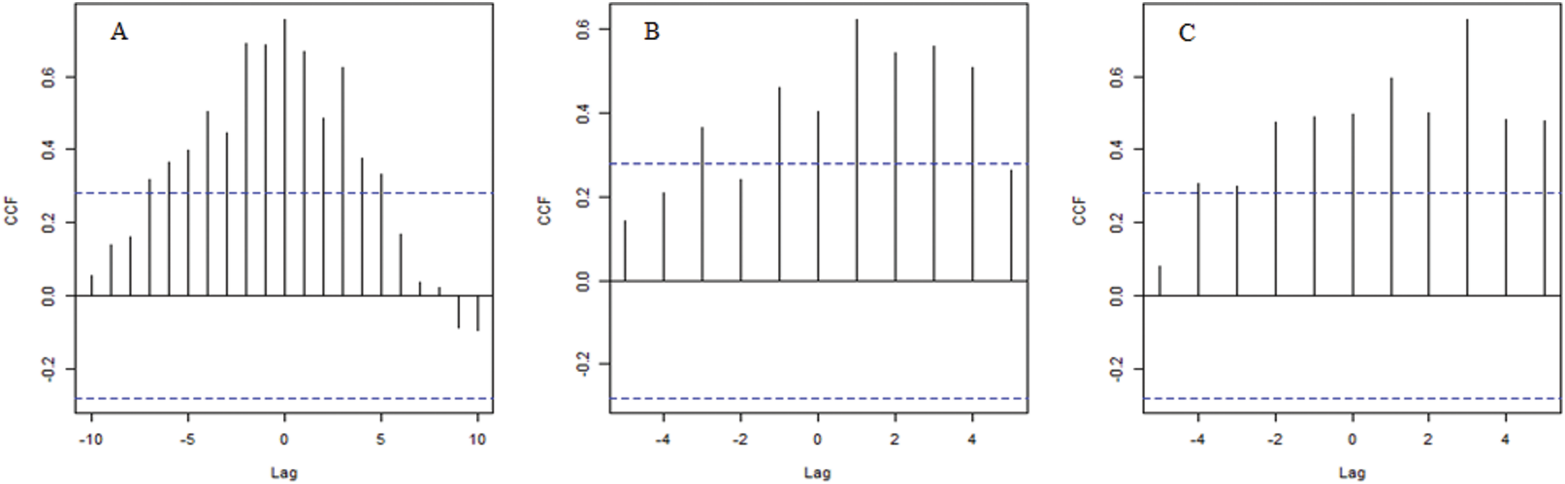
CCF among different lags of weeks. (A) CCF between egg abundance (total number of eggs/number of ovitraps) from both localities; the CCF shows significance from a time distance of 0 to 7 wk; (B) CCF between egg abundance and rainfall in TAH (B) and SPC (C), rainfall leads egg abundance, and has a positive influence in mosquito abundance. Dashed lines indicate the 95% confidence bounds.

CCFs using weekly mean egg collections for 2017 showed high positive cross-correlation between egg abundances from both localities with the strongest correlation found when no temporal lag was considered (lag = 0) (Fig. 2A). A significant positive cross-correlation between eggs abundance and pluvial precipitation were observed in TAH, with maximum correlation registered (CFC = 0.46) 1 wk prior the rain event (Fig. 2B). For SPC, the peak in cross-correlation occurred equally for a lag of zero or 1–2 wk before the rain (Fig. 2C).

### Oviposition

**Fig. 3.**
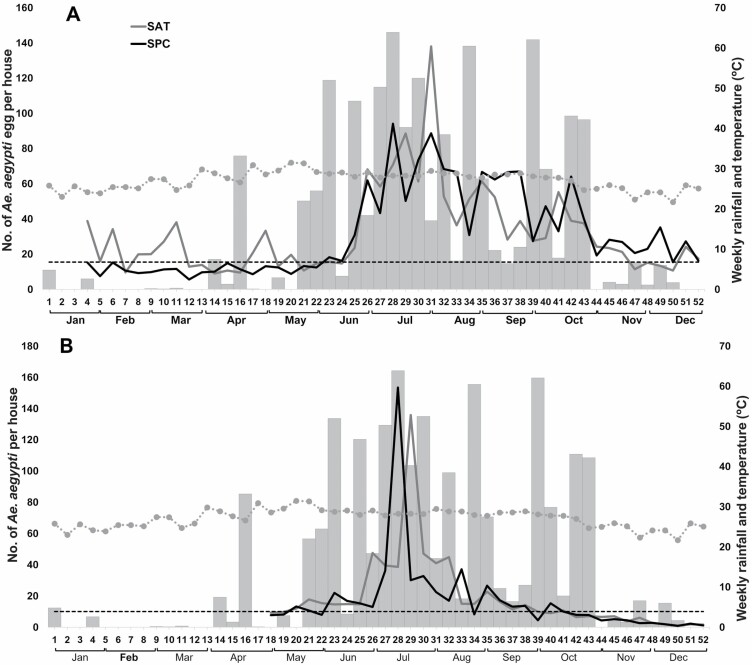
(A) Average number of *Ae. aegypti* eggs per total ovitraps/house per week; (B) average number of adult *Ae. aegypti* (females and males) collected in 24-h cycles at BG traps/houses per week and average temperature (dotted gray line) and monthly rainfall (gray bars) during 2017 in SPC and TAH. The dotted horizontal line represents the weekly average of *Aedes* abundance for both localities during the dry season (W1–W20).

The weekly average number of *Ae. aegypti* eggs collected from January to December 2017 was 31.69 ± 0.76 (mean 31.46, 95% CI: 29.46–35.19 for SPC and 31.92, 95% CI: 29.73–34.11 for TAH). During the dry season (W1–W20), the EDI, in both localities, showed its lowest level, never reaching 40 eggs per ovitrap per house (weekly average of 10.91, 95% CI: 9.41–12.42 for SPC and 19.94, 95% CI: 17.65–22.23 for TAH) (Fig. 3A). EDI showed an incremental growth from W24 and reached its peak between W27 and W32 (mean 69.55, 95% CI: 60.76–78.33 for SPC and 78.75, 95% CI: 66.49–91.01 for TAH) (Fig. 3A). After W35–38, EDI showed a monotonic decrease, returning to low abundance levels (<30 eggs per ovitrap per house) between W44 and W52 (mean 23.59, % CI: 20.64–26.54 for SPC and 18.18, 95% CI: 15.76–20.59 for TAH) (Fig. 2A).

### Adult Abundance

The 24-h ADI across both towns was 18.36 ± 0.88 individuals (95% CI: 16.64–20.08) per trap per house (mean 17.11, 95% CI: 15.05–19.17 for SPC and 19.64, 95% CI: 16.87–22.42 for TAH) with an average ADI of 11.48 ± 0.56 females (95% CI: 10.38–12.57), 7.62 ± 0.41 blood-fed females (95% CI: 6.81–8.42), and 6.88 ± 0.37 males (95% CI: 6.15–7.61) per house from May to December 2017 (Fig. 3B). A narrower, but similar, temporal trend was observed for ADI than for EDI throughout the year (Fig. 3), with average ADI during the dry season averaging 9.7 for SPC and 10.4 for TAH.

### Male Abundance

**Fig. 4.**
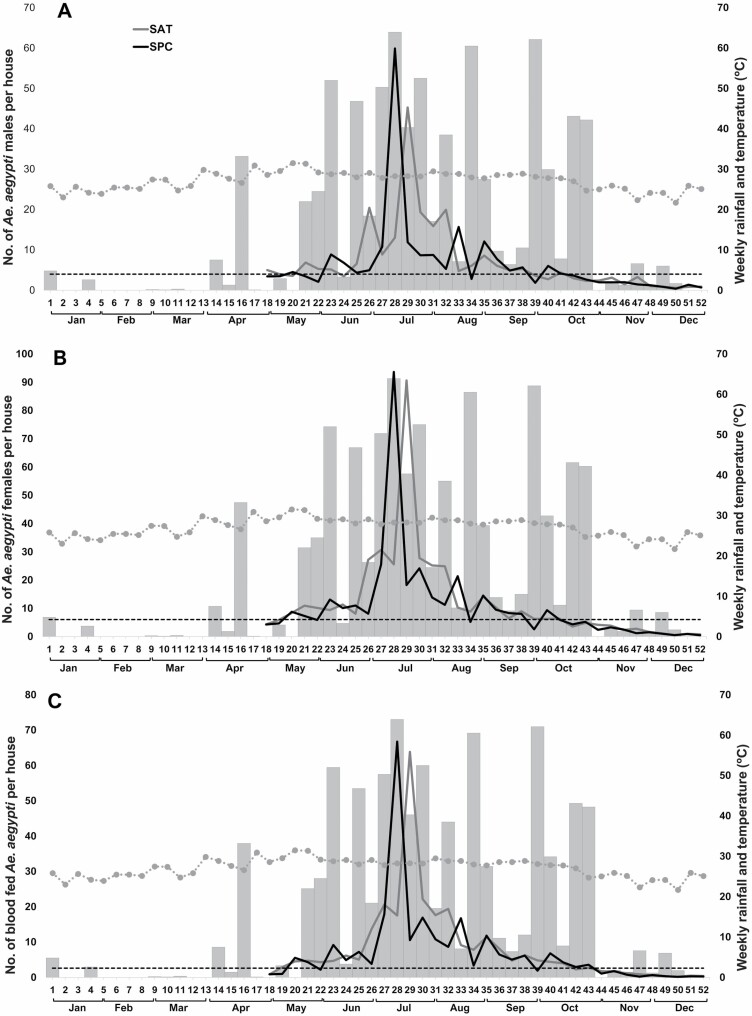
(A) Average number of adult *Ae. aegypti* males, (B) *Ae. aegypti* females, and (C) blood-fed females collected in 24-h cycles at BG traps/houses per week and average temperature (dotted gray line) and monthly rainfall (gray bars) from May to December 2017 in SPC and TAH. The dotted horizontal line represents the weekly average of *Aedes* abundance for both localities during the dry season (W1–W20).

The ADI for male *Ae. aegypti* was 6.88 ± 0.37 (95% CI: 6.15–7.61) males per house (mean 6.49, 95% CI: 5.63–7.36 for SPC and 7.28, 95% CI: 6.09–8.47 for TAH) from May to December 2017 (Fig. 4A). ADI for males was less than five males per house during the dry season (weekly average of 3.83, 95% CI: 3.05–4.60 for SPC and 4.19, 95% CI: 3.02–5.37 for TAH) (Fig. 4A). Male abundances increased noticeably when the rainy season started (W25), with a pronounced increment that reached a peak between W27 and W30 at both towns. At their highest level of abundance, the average number of male *Ae. aegypti* reached >40 individuals per trap/house/24 h (mean 22.93, 95% CI: 17.34–28.52 for SPC and 21.24, 95% CI: 12.65–29.83 for TAH) (Fig. 4A). After W30, constant decreases in the abundance of male adults were observed, and the abundances of *Ae. aegypti* males in both localities returned to low abundances (mean 1.34, 95% CI: 1.07–1.62 for SPC and 1.60, 95% CI: 1.23–1.96 for TAH between W44 and W52), similar to those observed during the dry season.

### Female Abundance

The average ADI during the dry season (Fig. 4B and C) was less than nine females (mean 5.97, 95% CI: 4.59–7.35 for SPC and 6.14, 95% CI: 4.14–8.14 for TAH) and less than six blood-fed females per house (mean 2.53, 95% CI: 1.66–3.40 for SPC and 2.72, 95% CI: 1.31–4.14 for TAH) (Fig. 4B and C). A pronounced increment in the abundance of *Ae. aegypti* females was observed from W25 and reached a peak between W27 and W30 in both towns (mean 40.62, 95% CI: 31.86–49.39 for SPC and 43.02, 95% CI: 31.27–54.78 for TAH). At their highest level of abundance, ADI reached >90 females (mean 93.62, 95% CI: 72.43–114.81 for SPC and 90.64, 95% CI: 51.15–130.12 for TAH) and >60 blood-fed females (mean 66.75, 95% CI: 50.69–82.81 for SPC, and 63.82, 95% CI: 33.99–93.64 for TAH) per house (Fig. 4B and C). After W30, there were constant decreases in the number of *Aedes* females per household. At the end of the year, the abundance of adult females in both localities returned to low levels of abundance (mean 1.58, 95% CI: 1.29–1.86 for SPC, and 2.00, 95% CI: 1.64–2.37 for TAH between W44 and W52), as observed during the dry season.

Three distinct periods/phases can be identified after the entomological collections, with a close association between precipitation and abundance of *Aedes* populations: 1) a phase of low population abundance among *Ae. aegypti* (weekly average of *Aedes* eggs and adults per trap = 15.51 ± 0.71 and 10.07 ± 0.88, respectively) during the dry season (mean 2.65 ± 1.67 mm of rain), which extended from January to the end of May (W1–W20); 2) a phase of population growth and the greatest relative abundance among *Aedes* (49.03 ± 1.48 eggs per ovitrap and 25.69 ± 1.31 *Aedes* adults per BG trap) during the rainy season (32.02 ± 4.14 mm), which extended from the end of May to October (W21–W43); and finally 3) a phase of decline among *Aedes* populations (20.91 ± 0.97 eggs per ovitraps and 3.24 ± 0.21 *Aedes* adults per BG trap) during the last part of the year and after the peak of the rainy season. This period is characterized by scattered rains (2.05 ± 0.84 mm) and extends from November to December (W44–W52).

## Discussion

We present results of a baseline characterization of *Ae. aegypti* abundance and seasonality in two localities that will be subject to a pilot study evaluating the IIT/SIT technique utilizing X-ray irradiation and *Wolbachia*-infected male *Ae. aegypti*. The seasonal abundance and dynamics of *Ae. aegypti* populations suggest that it is feasible to develop and implement time-specific actions that capitalize on *Ae. aegypti* seasonality as part of an IVM approach incorporating IIT–SIT for *Ae. aegypti* control in both suburban localities of Yucatan.

Comparability of population abundance and dynamics are parameters and inclusion criteria for the selection of appropriate sites for pilot trials (e.g., intervention and control) or initial implementation studies for population suppression (World Health Organization and International Atomic Energy Agency 2020). At both study sites, *Ae. aegypti* abundance/density was similar for both ovitrap and adult indices, and population dynamics were also similar (did not vary between sites). In addition to biological–ecological parameters, other inclusion criteria at these study sites are that both localities are convenient in size, e.g. small suburban towns (30–50 ha) that are far from large urban centers and are separated from each other but share similar ecological environments; they are similar in sociodemographic characteristics, e.g., human population density, housing, services, and infrastructure; and, last but not least, they do not have ongoing reports of Aedes-borne diseases (ABD) cases (Pan American Health Organization 2019, Bouyer et al. 2020, World Health Organization and International Atomic Energy Agency 2020).

The timing of the rainy season is an important indicator for determining the optimal pattern of male releases and the complementary scheduling of traditional vector control tools. Previous studies in Yucatan have noted a marked and very seasonal pattern of abundance of *Ae. aegypti* populations (Manrique-Saide et al. 2008, 2014; García-Rejón et al. 2011; Vazquez-Prokopec et al. 2017; Cauich-Kumul et al. 2018; Koyoc-Cardeña et al. 2019; González-Olvera et al. 2021). Correspondingly, vector control activities of the MoH of Yucatan (and Mexico) are typically programmed considering the occurrence of the rainy season as a traditional reference for vector control activities and a predictor for mosquito abundance and the consequent risk for ABD transmission (Diario Oficial de la Federación 2015).

As part of conventional activities before the rainy season, the vector control program of Yucatan usually organizes and performs clean-up campaigns (*descacharrización*); truck-mounted ULV is also commonly performed in response to increased risk of transmission suggested by increases in mosquito numbers which commonly occur during the rainy season (Diario Oficial de la Federación 2015). Therefore, in consensus with the MoH, we propose that anticipatory control activities affecting the mosquito population, such as clean-up campaigns and truck-mounted ULV, can be applied before the increases of mosquito populations/start of the rainy season (Fig. 4). Subsequently, these actions can be followed by *Wolbachia*-carrying male releases for an IVM strategy integrating the use of *Wolbachia*-based population suppression with traditional control.

**Fig. 5.**
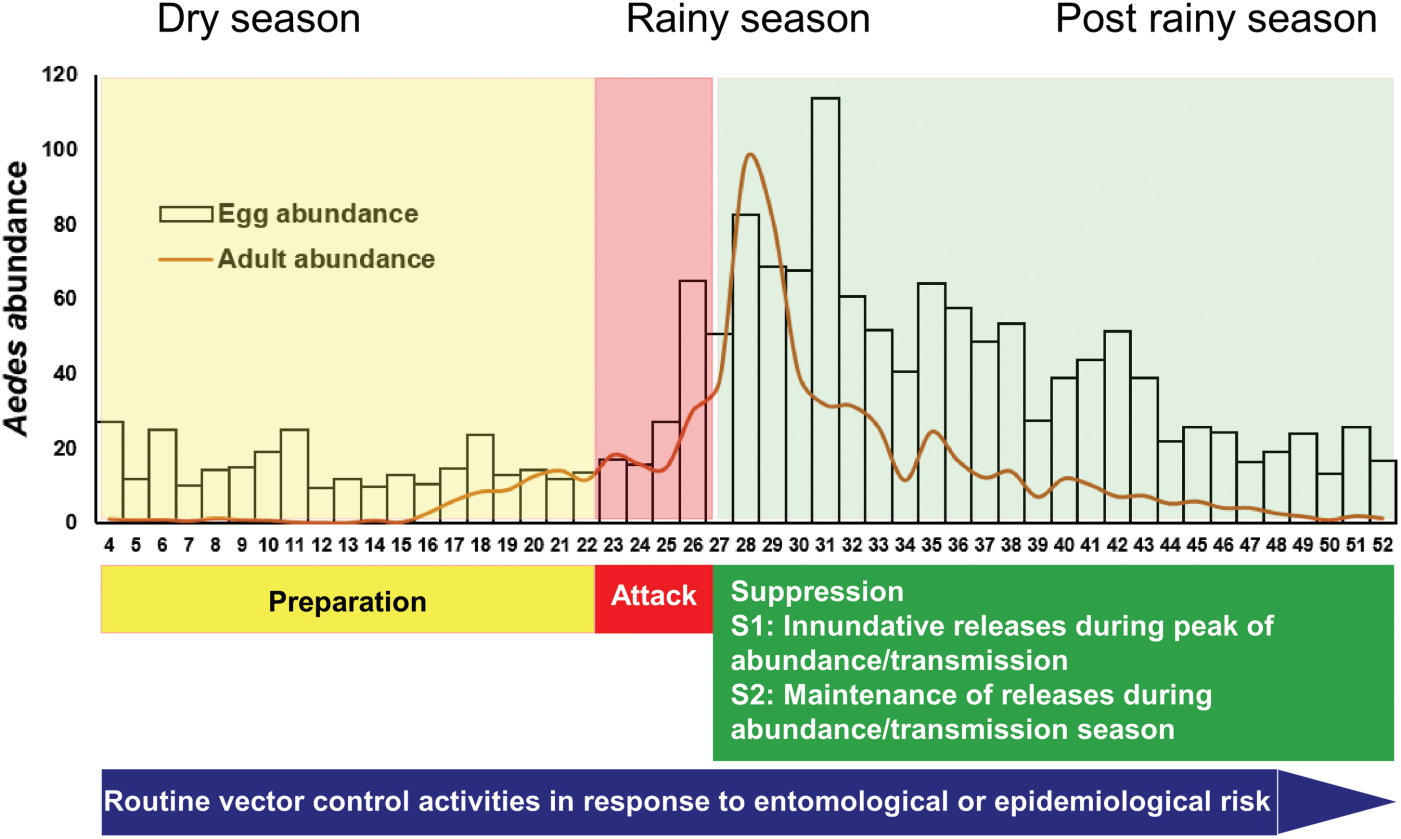
Proposed pilot IVM plan combining ‘traditional *Aedes* control’ and IIT/SIT based on the release of X-ray irradiated male *Ae. aegypti* carrying *Wolbachia* for population suppression. The strategy will be structured in three phases: Preparation: with community sensitization and engagement, and baseline entomological studies; Attack: initial traditional vector control; and Suppression phase: S1: inundative releases during peak of abundance/transmission and S2: maintenance of releases during abundance/transmission. Curves of egg and adult abundances are based on the average results from both study sites.

Thus, and in agreement with the vector control program of the Yucatan MoH, we initially propose a pilot IVM strategy structured in three phases (Fig. 5): 1) preparation phase (W1–20): aimed to complete social support through community education (e.g., workshops), sensitization, and engagement of local stakeholders for the implementation of the *Ae. aegypti* IVM strategy. Baseline (preintervention) entomological monitoring using ovitraps and BG-S baited traps will also be started. In this phase, and prior to the entomological surveys, towns will be divided in sectors of 1 ha to guide where sampling and mosquito releases will occur (Fig. 1C); 2) attack phase (W21–W27) with the implementation of traditional vector control actions: a) elimination of potential breeding habitats of immature mosquitoes through solid waste disposal campaigns (clean-up campaigns) with community participation and elimination of productive breeding sites (also to be implemented during the rest of the year by the community); followed by b) chemical control of adult *Aedes* (malathion outdoor ULV fogging) in four weekly applications covering the entire locality (W24–W27); and 3) suppression phase (W28–W52): with S1: inundative releases during peak of abundance/transmission and S2: maintenance of releases during abundance/transmission season. Releases of *Ae. aegypti Wolbachia*-carrying males twice a week (with 2-d intervals to avoid complaints related to an excess of mosquitos). Release of *Wolbachia*-carrying males can start from W28 and continue for 6 mo (July–December), covering the rainy season and the period of highest abundances of local *Ae. aegypti* populations.

The early characterization and quantification of parameters, such as abundance/density and seasonality of natural *Ae. aegypti* populations, are also important to calculate release ratios (released males:wild males) and to confirm feasibility (Lees et al. 2015, Bouyer et al. 2020). For our study sites, the number of *Aedes* males to be released can be adjusted based on a minimal target ratio from 5:1 to 10:1 (Kittayapong et al. 2019, Zheng et al. 2019). Previous research suggests that BG-Sentinel traps at a density of one per 15 houses capture around 5–10% of the adult population per week (Ritchie et al. 2013). Based on the maximum number of male *Aedes* calculated during the rainy season of 2017, and assuming that the efficiency of BG-S traps in Yucatan is comparable with those collected in northern Australia, we estimated a density of 168 males (maximum of males collected in a BG trap at one premise in 24 h × 10) per hectare (the average of houses per hectare in the intervened site is six houses per hectare, much less than 15 premises reported for Australia). This calculation implies that for each 1-ha area and for release ratios of 5:1 to 10:1 (released males:wild males), we would need to release from 1,000 to 2,000 males per hectare, and 30,000–60,000 males for an entire town (30 ha). We are considering releasing *Aedes* males twice a week—with an interval of 2 days between releases—meaning 60,000–120,000 males per week will need to be produced for the entire town per week. These figures are consistent with previous studies involving sterile male releases (Neira et al. 2014, Carvalho et al. 2015, Kittayapong et al. 2019, Mains et al. 2019).

**Fig. 6.**
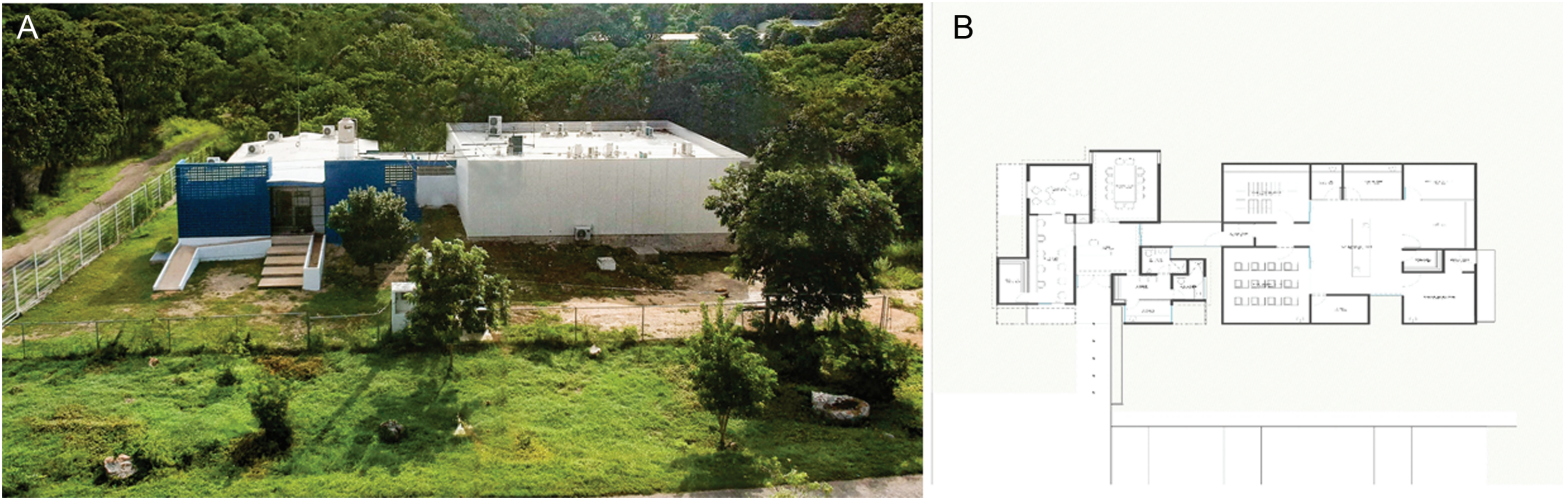
(A) Laboratory for biological control of *Ae. aegypti* of the Autonomous University of Yucatan (LCB-UADY) and (B) architectonic design details of the facility.

Thus, from an operational point of view, the Laboratory for Biological Control of *Ae. aegypti* of the UADY (LCB-UADY) will need to produce and provide to the MoH at least 250,000 males (an extra production always must be considered additional to required needs) (Zheng et al. 2019) to be transported and delivered in adequate containers (with 1,000 males per container) for open-field releases. This production is feasible to achieve at the LCB-UADY. Inaugurated on September 2018, the LCB-UADY has an installed capacity to produce 3–5 million *Ae. aegypti* males per week (Fig. 6). Premises, equipment, and production processes follow, at a reduced scale, those established at the Joint Center of Vector Control for Tropical Diseases SYS-MSU (Zhang et al. 2015, 2016; Zheng et al. 2019).

Entomological surveillance for an IVM approach incorporating IIT–SIT for *Ae. aegypti* control with releases of incompatible/sterile males will require monitoring and assessment of the different interventions/components to quantify the level of suppression achieved (World Health Organization and International Atomic Energy Agency 2020). Therefore, a combination of trapping methods for different life stages is needed for entomological surveillance. Current initiatives for *Aedes* population suppression have used and recommend ovitrapping, gravitraps, or other adult traps, targeting both adult male and female mosquitoes, which represent the epidemiologically important target (Carvalho et al. 2015, Kittayapong et al. 2019, Mains et al. 2019, World Health Organization and International Atomic Energy Agency 2020).

Changes in the intensity of oviposition such as reductions of number of eggs can be associated with decreases in the total population (Tantowijoyo et al. 2016, Kittayapong et al. 2019, Mains et al. 2019, Zheng et al. 2019); importantly, they can be very sensitive even at very low abundance levels (for example, when population suppression is successful). In addition, and particularly applicable for monitoring the population suppression with IIT/SIT method, egg hatching (or no-hatching) of material collected from ovitraps is a measure of the success of incompatible/sterile males, shown by the proportion infertile eggs collected at ovitraps (Mains et al. 2019, Zheng et al. 2019). BG-sentinel traps collect adults (both males and females) outdoors in the vicinity of the trap and have previously been used in assessing population size at a local scale (Ritchie et al. 2013). During male releases, BG-sentinel traps can be useful for monitoring adult mosquitoes and their population changes, and even more, can provide specimens to be screened for viruses.

Any country considering the implementation of strategies with releases of incompatible/sterile-male *Aedes* mosquitoes will also have to think about modifying and augmenting the capabilities of its entomological surveillance system. An enhanced entomological surveillance system is indispensable; in addition to, of course, an epidemiological surveillance system with the ability to monitor spatial, temporal, and pre–post impact changes (Pan American Health Organization 2019). Our study provides the foundation for the pilot evaluation of IIT/SIT in Mexico and highlights the value that local vector control agencies will see in integrating novel technologies (such as rear-and-release of sterile/incompatible males) with classic (insecticide-based) approaches implemented routinely for vector control.
